# Genome Dynamics of Vibrio cholerae Isolates Linked to Seasonal Outbreaks of Cholera in Dhaka, Bangladesh

**DOI:** 10.1128/mBio.03339-19

**Published:** 2020-02-11

**Authors:** Ramani Baddam, Nishat Sarker, Dilruba Ahmed, Razib Mazumder, Ahmed Abdullah, Rayhan Morshed, Arif Hussain, Suraiya Begum, Lubaba Shahrin, Azharul Islam Khan, Md Sirajul Islam, Tahmeed Ahmed, Munirul Alam, John D. Clemens, Niyaz Ahmed

**Affiliations:** a International Centre for Diarrhoeal Disease Research, Bangladesh (icddr,b), Dhaka, Bangladesh; KUMC

**Keywords:** *Vibrio cholerae*, genomics, seasonality, serogroups

## Abstract

The temporal switching of serotypes from serotype Ogawa to Inaba and back to Ogawa was identified in Vibrio cholerae O1, which was responsible for seasonal outbreaks of cholera in Dhaka during the period 2015 to 2018. In order to delineate the factors responsible for this serotype transition, we performed whole-genome sequencing (WGS) of V. cholerae O1 multidrug-resistant strains belonging to both the serotypes that were isolated during this interval where the emergence and subsequent reduction of the Inaba serotype occurred. The whole-genome-based phylogenetic analysis revealed clonal expansion of the Inaba isolates mainly responsible for the peaks of infection during 2016 to 2017 and that they might have evolved from the prevailing Ogawa strains in 2015 which coclustered with them. Furthermore, the *wbeT* gene in these Inaba serotype isolates was inactivated due to insertion of a transposable element at the same position signifying the clonal expansion. Also, V. cholerae isolates in the Inaba serotype dominant clade mainly contained classical *ctxB* allele and revealed differences in the genetic composition of *Vibrio* seventh pandemic island II (VSP-II) and the SXT integrative and conjugative element (SXT-ICE) compared to those of Ogawa serotype strains which remerged in 2018. The variable presence of phage-inducible chromosomal island-like element 1 (PLE1) was also noted in the isolates of the Inaba serotype dominant clade. The detailed genomic characterization of the sequenced isolates has shed light on the forces which could be responsible for the periodic changes in serotypes of V. cholerae and has also highlighted the need to analyze the mobilome in greater detail to obtain insights into the mechanisms behind serotype switching.

## Abstract (precis)

The switching of serotype from Ogawa to Inaba and back to Ogawa has been observed temporally in Vibrio cholerae O1, which is responsible for endemic cholera in Bangladesh. The serospecificity is key for effective intervention and for preventing cholera, a deadly disease that continues to cause significant morbidity and mortality worldwide. In the present study, WGS of V. cholerae allowed us to better understand the factors associated with the serotype switching events observed during 2015 to 2018. Genomic data analysis of strains isolated during this interval highlighted variations in the genes *ctxB*, *tcpA*, and *rtxA* and also identified significant differences in the genetic content of the mobilome, which included key elements such as SXT ICE, VSP-II, and PLE. Our results indicate that selective forces such as antibiotic resistance and phage resistance might contribute to the clonal expansion and predominance of a particular V. cholerae serotype responsible for an outbreak.

## INTRODUCTION

Cholera, a deadly waterborne diarrheal illness caused by toxigenic Vibrio cholerae species, is more prevalent in developing countries such as Bangladesh due to use of unsafe drinking water with poor sanitation (1). The burden of cholera is very high in Bangladesh, with an estimated ∼100,000 cases reported annually and close to 4,500 deaths per year (2). V. cholerae includes more than 200 serogroups identified to date, of which only the O1 and O139 serogroups are responsible for the cholera outbreaks (3). Globally, however, the O1 serogroup remains the primary cause of cholera and is distributed into two biotypes, namely, classical and El Tor, which differ in their biochemical properties and susceptibility to certain phages (4). The classical biotype of V. cholerae surmised to be responsible for the first six pandemics was completely replaced by the El Tor biotype associated with the ongoing seventh pandemic, which began in early 1960s. This El Tor biotype originated in Asia and was subsequently found to have distributed all over the world in the form of three phylogenetically distinct but overlapping waves (5). Remarkably, the typical El Tor biotype strains can persist under different environmental conditions and display dynamic host-to-host transmission compared to classical biotype strains. However, classical biotype strains produce a cholera toxin (CT) that is responsible for serious clinical outcomes (6).

In the 1990s, atypical variants (most likely because of horizontal gene transfer [HGT] between biotypes) of V. cholerae biotype El Tor strains were identified in Bangladesh and were shown to exhibit combined biological features; these El Tor variant strains also contained a El Tor or classical CTXφ prophage in their genomes and characteristically produced classical cholera toxin (7–10). Further, acquisition of the SXT integrative and conjugative element (ICE), which conferred important antibiotic resistance phenotypes, contributed to the infectious potential of V. cholerae O1 El Tor variants (11, 12).

The biotypes of the O1 serogroup can be further classified into serotypes—mainly, Ogawa and Inaba, both capable of causing severe cholera characterized by dehydrating diarrhea (4). These serotypes can be differentiated by the methylation status of the surface lipopolysaccharides (LPS), and the methyltransferase enzyme which catalyzes this reaction is encoded by the *wbeT* gene (previously called *rfbT*) (13). Strains with methylated LPS are identified as Ogawa serotype strains, whereas strains with nonmethylated LPS due to inactivation of the enzyme by mutations in *wbeT* gene are identified as Inaba serotype strains (14). The serotype conversion or switching between these two serotypes has been documented in various parts of the world, including Bangladesh (15–18). The genetic changes in the isolates belonging to these two serotypes were previously studied using various molecular typing techniques (19–21). Using whole-genome sequencing (WGS), a retrospective analysis of V. cholerae isolates belonging to both serotypes was conducted previously in a zone of cholera endemicity (Kolkata, India) to understand the serotype switching (22). Although that study identified genetic changes in the *wbeT* gene that led to disruption of the methyltransferase enzyme, the results indicated that such serotype switching is not random; the mechanisms behind these phenomena are yet to be identified. Collectively, various studies have highlighted the importance of close monitoring of serotype switching/cycling in V. cholerae and the need to understand the factors behind this phenomenon.

In the current study, we examined the serotype trends of cholera cases reported to the International Centre for Diarrhoeal Disease Research, Bangladesh (icddr,b), from 2005 to 2018. Further, from the trends gleaned, it was noted that nearly complete replacement of the Ogawa serotype by the Inaba serotype occurred during the period 2016 to 2017. In order to delineate the factors behind this serotype transition, we performed whole-genome sequencing of 34 strains belonging to both serotypes isolated during this interval (2015 to 2018), where emergence and subsequent reduction of Inaba serotype had occurred. We hypothesized that detailed genomic characterization of these isolates would shed light on the selective forces which might be responsible for the periodic changes in serotypes of V. cholerae in Bangladesh. The results of this study emphasize the importance of analyzing the whole-genome sequences of both serotypes, especially the mobilome, in order to gain new insights into the mechanisms responsible for the intermittent upsurge of different serotypes.

## RESULTS

### Seasonality and serotype distribution of cholera outbreaks.

**FIG 1:**
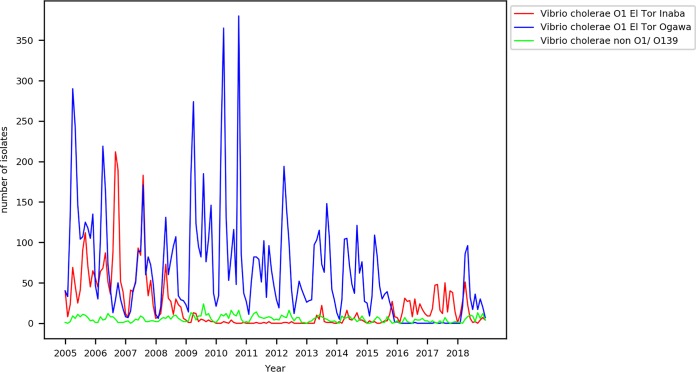
Temporal distribution of Vibrio cholerae serotypes from 2005 to 2018. The graph shows the serotype distribution of V. cholerae isolates identified at icddr,b, Dhaka, Bangladesh, for a 14-year period (2005 to 2018). The Ogawa and Inaba serotypes of V. cholerae are indicated in blue and red, respectively. The non-O1/O139 serotypes are represented in green.

The metadata of V. cholerae cases referred to the Clinical Microbiology and Immunology Laboratory of icddr,b for bacterial isolation and confirmation were gleaned for a period of 14 years (2005 to 2018). The seasonality of cholera in this area of endemicity is reflected in the form of biannual peaks, usually occurring before and after the rainy season as also noted previously (23, 24). As shown in Fig. 1, for first 4-year period (i.e., between 2005 and 2008), isolates belonging to both serotypes were identified during the periodic outbreaks, although in differing numbers. However, beginning from 2009, remarkably, the peaks of infection were due only to Ogawa cases and the numbers of Inaba cases were found to have been significantly reduced until 2015. Inversely, the peaks of infection for the years 2016 and 2017 were due only to Inaba cases and the number of Ogawa cases began to increase again only from the beginning of 2018. Further, exceptionally low numbers of total cholera cases were reported in these 2 years (2016 and 2017) compared to the whole period of 14 years (2005 to 2018). In general, Ogawa serotype isolates were dominant for the majority of the time period analyzed, although a striking predominance of the Inaba serotype was observed in the second half of 2006, and, after roughly a decade, in the years 2016 to 2017, almost near replacement of Ogawa serotype cases by Inaba serotype cases was observed. The rates of identification of Ogawa and Inaba cases during the transition period 2015 to 2018 are depicted (with an interval corresponding to each month) in Fig. S1 in the supplemental material.

10.1128/mBio.03339-19.1 FIG S1 Temporal distribution of Vibrio cholerae serotypes from 2015 to 2018. The data represent the rate of isolation of V. cholerae Ogawa and Inaba serotypes at each month’s interval at icddr,b, Dhaka, Bangladesh, during the study period 2015 to 2018. Ogawa and Inaba serotypes of V. cholerae are indicated in blue and red, respectively. Download FIG S1, TIF file, 0.4 MB. Copyright © 2020 Baddam et al. 2020 Baddam et al. This content is distributed under the terms of the Creative Commons Attribution 4.0 International license.

### Genomic features and phylogenetic analysis.

In order to understand the mechanisms behind this serotype switching, we performed WGS of 34 V. cholerae isolates belonging to both the serotypes (Inaba and Ogawa) isolated between the years 2015 and 2018, during which emergence and subsequent reduction of Inaba serotype had occurred. The associated metadata of isolates, along with their genome features and accession details, are listed in Table 1. The depth of sequencing coverage of isolates ranged from 32× to 121×, with a median value of 73×. The draft genome sizes of V. cholerae isolates approximately ranged from 3.9 to 4.0 Mbp, with average G+C content of 47.45%. All of these isolates belonged to the seventh pandemic El Tor biotype. The details of the antibiotic sensitivity test results are provided in Table 2. All 34 V. cholerae isolates were multidrug resistant and exhibited resistance to at least three or more classes of antibiotics, and all of them were sensitive to only a single antibiotic—azithromycin. Moreover, 33 out of 34 V. cholerae isolates were also resistant to ampicillin, nalidixic acid, furazolidone, nitrofurantoin, streptomycin, and polymyxin B.

**TABLE 1:** Genome features and metadata of 34 V. cholerae isolates in this sequenced study^*a*^

Sl no.	Isolate ID	Date of isolation	Serotype	Accession no.	Cov	Contig no. (≥200 bp)	Genome size (bp)	No. of CDS	Status of *wbeT* gene
1	NALMLE01	June 2018	Ogawa	SIUC00000000	42×	126	4,024,523	3,526	NC
2	NALMLE02	June 2018	Ogawa	SIUD00000000	56×	109	4,039,991	3,545	WT
3	NALMLE03	July 2018	Ogawa	SIUE00000000	63×	93	4,038,801	3,643	WT
4	NALMLE05	July 2018	Ogawa	SIUG00000000	54×	101	4,059,930	3,590	WT
5	NALMLE06	July 2018	Ogawa	SIUH00000000	43×	153	4,021,632	3,524	NC
6	NALMLE07	July 2018	Ogawa	SIUI00000000	40×	145	4,019,165	3,527	NC
7	NALMLE08	July 2018	Ogawa	SIUJ00000000	53×	134	4,027,137	3,532	NC
8	NALMLE11	August 2018	Ogawa	SIUM00000000	43×	155	4,014,513	3,518	NC
9	NALMLE13	August 2018	Ogawa	SIUO00000000	44×	148	4,008,985	3,510	NC
10	NALMLE15	August 2018	Ogawa	SIUQ00000000	43×	130	4,028,344	3,532	WT
11	NALMLE20	January 2017	Inaba	SIUV00000000	32×	137	4,043,029	3,571	Tran
12	NALMLE22	March 2018	Ogawa	SIUX00000000	110×	94	4,042,008	3,550	WT
13	NALMLE23	March 2018	Inaba	SIUY00000000	79×	88	3,945,614	3,473	Tran
14	NALMLE24	March 2018	Inaba	SIUZ00000000	95×	93	3,944,553	3,469	Tran
15	NALMLE25	April 2018	Ogawa	SIVA00000000	112×	86	4,041,480	3,552	WT
16	NALMLE26	April 2018	Ogawa	SIVB00000000	94×	81	4,041,012	3,553	WT
17	NALMLE27	April 2018	Ogawa	SIVC00000000	105×	89	4,040,960	3,550	WT
18	NALMLE28	April 2018	Inaba	SIVD00000000	102×	99	4,035,854	3,560	Tran
19	NALMLE29	April 2018	Inaba	SIVE00000000	77×	95	3,945,938	3,469	Tran
20	NALMLE30	May 2018	Ogawa	SIVF00000000	94×	89	4,042,613	3,554	WT
21	NALMLE31	May 2018	Ogawa	SIVG00000000	71×	88	4,041,829	3,549	WT
22	NALMLE33	April 2017	Inaba	SIVI00000000	68×	105	4,038,540	3,558	Tran
23	NALMLE34	April 2017	Inaba	SIVJ00000000	65×	92	4,037,260	3,560	Tran
24	NALMLE35	April 2017	Inaba	SIVK00000000	75×	88	4,055,692	3,588	Tran
25	NALMLE36	May 2017	Inaba	SIVL00000000	91×	89	4,055,930	3,595	Tran
26	NALMLE37	May 2017	Inaba	SIVM00000000	43×	92	4,037,271	3,559	Tran
27	NALMLE38	May 2017	Inaba	SIVN00000000	55×	81	4,017,050	3,541	Tran
28	NALMLE39	April 2016	Inaba	SIVO00000000	90×	87	4,055,742	3,589	Tran
29	NALMLE40	April 2016	Inaba	SIVP00000000	99×	90	4,056,494	3,590	Tran
30	NALMLE41	May 2016	Inaba	SIVQ00000000	79×	99	4,055,425	3,586	Tran
31	NALMLE42	April 2015	Inaba	SIVR00000000	121×	100	4,062,426	3,691	Tran
32	NALMLE43	May 2015	Ogawa	SIVS00000000	48×	110	4,062,040	3,587	WT
33	NALMLE44	May 2015	Ogawa	SIVT00000000	90×	87	4,043,623	3,568	WT
34	NALMLE45	May 2015	Ogawa	SIVU00000000	83×	85	4,056,579	3,594	WT

a Sl no., serial number; ID, identifier; Cov, genome coverage; CDS, coding DNA sequences; NC, gene not covered completely; WT, wild type; Tran, disrupted due to transposable elements.

**TABLE 2:** Antibiotic susceptibility profiles of 34 V. cholerae isolates in this sequencing study

Sl no.	Antibiotic	No. (%) of resistant isolates
1	Ampicillin	34 (100)
2	Azithromycin	0
3	Nalidixic acid	33 (97)
4	Tetracycline	15 (44)
5	Chloramphenicol	14 (41)
6	Ciprofloxacin	15 (44)
7	Furazolidone	33 (97)
8	Norfloxacin	5 (14)
9	Co-trimoxazole	30 (88)
10	Erythromycin	5 (14)
11	Nitrofurantoin	33 (97)
12	Cefotaxime	20 (58)
13	Polymyxin B	34 (100)
14	Streptomycin	33 (97)
15	MDR^*a*^ phenotype	34 (100)

a MDR, multidrug resistant.

**FIG 2:**
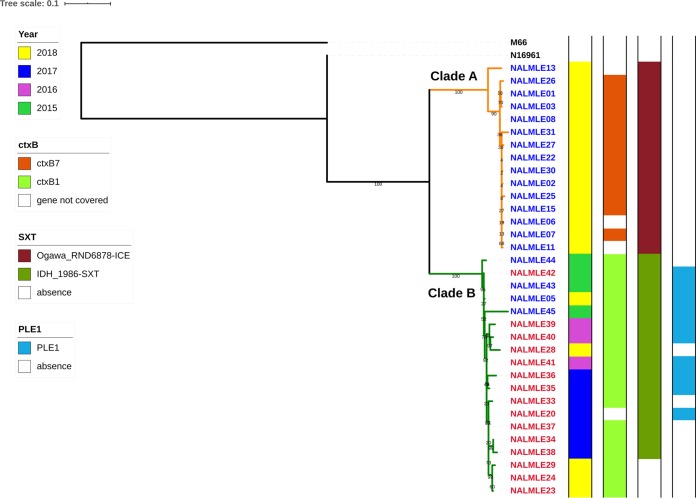
Whole-genome-based phylogeny. The whole-genome-based phylogenetic tree of 34 in-house-sequenced V. cholerae isolates along with genomes of reference strain N16961 and outgroup strain M66 revealed broad correlation between the year of isolation and serotype. The colors of the node labels indicate the serotypes as follows: blue, Ogawa; red, Inaba. Clades A and B are marked in orange and green, respectively. The adjacent colored bars indicate the year of isolation, *ctxB* allele, SXT ICE (integrative conjugative element), and PLE1 (phage-inducible chromosomal island-like element 1), respectively.

A maximum likelihood phylogenetic tree generated from a whole-genome alignment of 34 in-house-sequenced genomes along with the genomes of reference strain N16961 and an outgroup isolate M-66 revealed two major clades—clade A and clade B—as shown in Fig. 2. In general, based on their clustering pattern, correlation between the year of isolation and the serotype can be observed. Clade A is comprised only of Ogawa serotype strains isolated during the year 2018. Although clade B included mainly Inaba serotype strains from 2015 to 2018, coclustering with three Ogawa serotype strains from 2015 and one isolate from 2018 was also seen.

From this analysis, it appears that clonal expansion of Inaba serotype isolates as seen in clade B was primarily responsible for the peaks of infection during 2016 to 2017 and that these isolates may have evolved from the prevailing Ogawa isolates of 2015 as indicated by the coclustering of isolates with Inaba dominant clade B. A similar clustering pattern was observed in the consensus tree built from an alignment of core genes identified using the Roary pan-genome pipeline (Fig. S2). The phylogenetic analysis revealed high genetic relatedness of isolates irrespective of their serotype identities (Ogawa and Inaba). Moreover, it was observed that all 34 V. cholerae isolates analyzed belonged to a single sequence type (ST)—ST 69. Genome-wide analysis of single nucleotide polymorphisms (SNPs) revealed that the 34 in-house-sequenced isolates differed from the reference genome of V. cholerae O1 El Tor strain N16961 by less than 160 SNPs after filtering those entailing recombination.

10.1128/mBio.03339-19.2 FIG S2 Core genome-based phylogeny. The core genome-based phylogenetic tree of 34 in-house-sequenced V. cholerae isolates along with genomes of reference strain N16961 and outgroup strain M66 is presented. The colors of the node labels indicate the serotypes as follows: blue, Ogawa; red, Inaba. The adjacent colored bars indicate the year of isolation, *ctxB* allele, SXT ICE (integrative conjugative element), and PLE1 (phage-inducible chromosomal island-like element 1). Download FIG S2, TIF file, 0.7 MB. Copyright © 2020 Baddam et al. 2020 Baddam et al. This content is distributed under the terms of the Creative Commons Attribution 4.0 International license.

The screening for polymorphisms in the *wbeT* gene, which encodes the methyltransferase enzyme responsible for methylation of LPS, was carried out to elucidate the serotype transition from Ogawa to Inaba. It was observed that the *wbeT* gene was disrupted due to insertion of a transposable element at the same position (nucleotides [nt] 398 to 399) in all Inaba isolates of clade B, except in the single Inaba serotype strain from 2015, where the gene was disrupted at a different position (nt 845 to 846). This observation further corroborates the clonal expansion of the Inaba isolates in clade B which were mainly responsible for the peaks of infection during 2016 to 2017. Furthermore, disruption of the gene at a different location in an Inaba serotype strain from the year 2015 indicates that the serotype switching observed in 2016 to 2017 cannot be explained by an Inaba index case. On the whole, it appears that Inaba serotype strains isolated during the next three years, 2016 to 2018, could have evolved from the prevailing Ogawa serotype isolates of 2015 which coclustered with Inaba serotype isolates in clade B of the phylogenetic tree (Fig. 2). The transposable element responsible for the *wbeT* gene disruption was similar to SXT IS4 family transposase gene (gb|KC709654.1), and it was reported previously (22). Also, no nonsynonymous mutations were identified as responsible for the inactivation of the *wbeT* gene.

In addition, the two clades revealed distinct differences in certain important virulence genes of V. cholerae. Clade A and clade B differed in the alleles of the *ctxB* gene, which encodes the B subunit of cholera toxin (CT); *ctxB1* or *ctxB^cla^* was mainly observed in Inaba serotype dominant clade B isolates, whereas *ctxB7* was mainly observed in Ogawa serotype isolates of clade A, and in case of few isolates, the gene was not covered completely as shown in Fig. 2. Also, key differences were observed in the *rtxA* gene which encodes a multifunctional autoprocessing RTX (MARTX) family toxin. Clade A Ogawa serotype isolates contained a nonfunctional *rtxA* gene due to a SNP (G to A) at nt 13602 of the gene which leads to introduction of a premature stop codon and loss of 12 amino acids (aa) at the C-terminal end as reported previously (25). Along with this SNP, a deletion of 60 bp from the position corresponding to nt 1734 to 1793 of the gene was also observed in all isolates of clade A. Altogether, these variations reduced the RtxA protein size from 4,545 aa to 4,513 aa, resulting in loss of 32 amino acids. In addition to the above-mentioned changes, an SNP (A to G) at nt position 9070 which resulted in the change of aa 3024 from serine to glycine and a deletion from nt 5348 to 5476 which further reduced the RtxA protein size to 4,470 aa were identified in the NALMLE13 and NALMLE31 strains of clade A, respectively.

In contrast to clade A, clade B, which is mainly composed of Inaba serotype isolates, contained a functional *rtxA* gene without any genetic variations except in the cases of two strains—NALMLE28 and NALMLE35. An SNP (A to C) at nt position 4319 which resulted in the change of aa 1440 from aspartic acid to alanine was observed in strain NALMLE28, and in the case of strain NALMLE35, the *rtxA* gene was split over two contigs, probably due to disruption by a transposable element similar to the SXT IS*4* family transposase gene. The characterization of alleles of the toxin coregulated pilus A gene (*tcpA*) revealed that a Haitian *tcpA* allele (tcpET^CIRS^) (26) was uniformly observed in all Ogawa and Inaba isolates sequenced in this study (except in the NALMLE15 strain, in which the gene is not covered completely).

### Comparative genomics and insights from the accessory genome.

**FIG 3:**
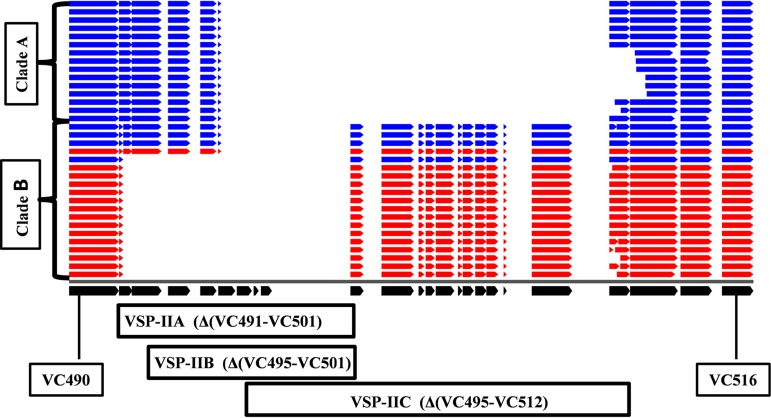
Genetic organization of VSP-II. The open reading frames (ORFs) of the VSP-II island (VC_490 to VC_516) in the genome of V. cholerae strain N16961 are represented in black at the bottom of the figure. The ORFs in each line in the top panel indicate their status in 34 in-house-sequenced V. cholerae isolates, and blue and red data indicate serotypes Ogawa and Inaba, respectively. The comparison was generated using BLAST Atlas.

The pan-genome analysis clearly highlighted variations in the accessory genome, mainly in hot spot regions such as the VSP-II, compared to V. cholerae reference strain N16961 of the seventh pandemic El Tor biotype. The genetic organization of this region in different isolates of our collection, as shown in Fig. 3, led to the identification of three main types broadly—VSP-IIA [Δ(VC_491 to VC_501)], VSP-IIB [Δ(VC_495 to VC_501)], and VSP-IIC [Δ(VC_495 to VC_512)]. Further, it was observed in a few isolates that the deletion at the right boundary was extended to either VC_513 or VC_514 in case of VSP-IIC and either to VC_500 or VC_501 in case of VSP-IIB. Further in depth analysis of the VSP-II region was affected by draft status of the genomes.

The VSP-IIA type was observed in all Inaba serotype isolates of clade B, except for one Inaba serotype strain NALMLE42 isolated in 2015 that harbored the VSP-IIB type. However, only the NALMLE45 Ogawa serotype strain from 2015 contained the VSP-IIA type, similarly to the majority of Inaba serotype isolates of clade B, whereas the remaining Ogawa serotype isolates which coclustered in clade B also contained the VSP-IIB type. The isolates in clade A exclusively contained VSP-IIC or its variants observed in a few isolates which differed in the right boundary. This analysis clearly indicated the genetic rearrangements in the VSP-II region of strains isolated during the transition period in 2015 and subsequent emergence of Inaba serotype isolates with the VSP-IIA type. However, the VSP-IIB type was also observed in strain NALMLE05 isolated in 2018, but it coclustered with clade B Ogawa and Inaba isolates.

Phage-inducible chromosomal island-like elements (PLEs) are specified genomic islands identified in V. cholerae whose main function is to provide the ability to defend against predatory phages (27). Interestingly, an 18,079-bp PLE1 island was observed in both Ogawa and Inaba serotype strains isolated in 2015 except in strain NALMLE44. Although all Inaba strains from the year 2016 harbored this element, only 3 of 7 Inaba isolates from the year 2017 possessed it. Further, beginning from 2018, this element was not observed in isolates of either of the serotypes, except in Ogawa serotype strain NALMLE05. The sequence of PLE1 identified in our strain collection was 99% to 100% similar to a previously published sequence (GenBank accession no. KC152960.1), and the majority of its genes encoded hypothetical proteins.

Further, in-depth analysis of accessory gene content revealed intriguing differences in the V. cholerae self-transmissible, chromosomally integrative and conjugative element (SXT ICE) that confers key antibiotic resistance phenotypes (12). Primarily, two different SXT ICEs were observed in isolates as shown in Fig. 2, which corresponds to the clustering of isolates into clades A and B. A ∼91-to-95-kbp SXT element similar to that present in strain IDH_1986 (GenBank accession MK165649.1) was identified in both Inaba and Ogawa serotype strains isolated in 2015. Further, this element was continuously present during the upheaval of the Inaba serotype during 2016 to 2017 and was noted to be present in all strains isolated during those 2 years. The loss of this particular SXT element in three of four Inaba isolates was first observed in 2018 when the Ogawa serotype reemerged. Another SXT element similar to the ICE of V. cholerae strain Ogawa RND6878 (GenBank accession KY382507.1) and ∼95 to 99 kbp in size was observed in isolates of clade A comprising exclusively Ogawa serotype strains from 2018. The only exception was Ogawa serotype strain NALMLE05; although isolated in 2018, it coclustered with Ogawa and Inaba isolates of clade B and contained an SXT element similar to that observed in strain IDH_1986.

### Mobile antibiotic resistance mediated by SXT ICE elements.

**FIG 4:**
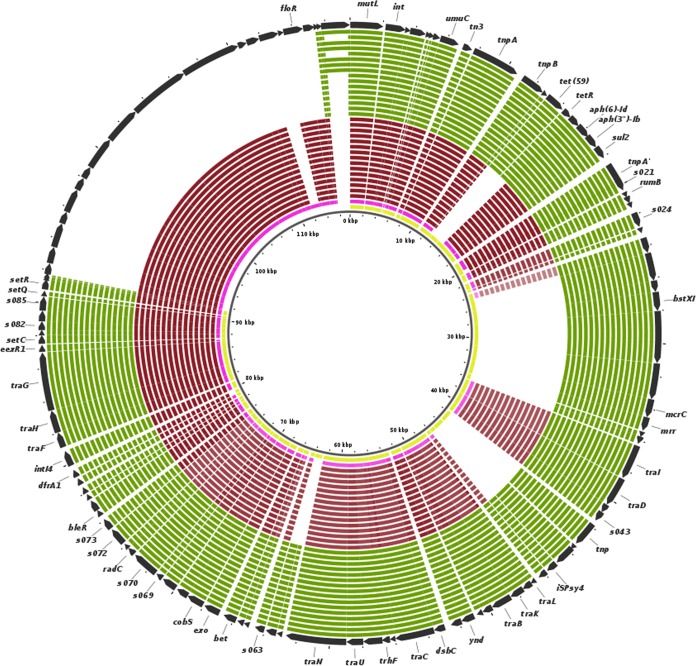
Variations in SXT ICE. The SXT pangenome was constructed iteratively by appending unique regions of SXT elements of 34 in-house-sequenced isolates and the Ogawa RND6878 strain onto that of the IDH_1986 strain which served as the initial seed. The SXT pangenome is represented in the outer circle (black), and the pink and yellow innermost circles represent individual SXT ICE elements of strains Ogawa RND6878 and IDH_1986, respectively. The red and green color coding of the rest of the inner circles indicates in-house-sequenced isolates belonging to clade A and clade B, respectively, as shown in Fig. 2.

SXT ICE elements are known to carry multiple antibiotic resistance genes and can be horizontally transferred between bacteria. The detailed comparison of resistance gene repertoires carried by two SXT ICE elements observed in our collection of isolates revealed that both carried genes for sulfonamide resistance (*sul2*), trimethoprim resistance (*dfrA1*), and streptomycin resistance [*aph(3*′′*)-Ib*, *aph(6)-Id*)]. An SXT ICE observed in isolates of clade B contained gene *tet*(59) conferring resistance to tetracycline as shown in Fig. 4, whereas an SXT ICE observed in isolates of clade A contained gene *floR* responsible for chloramphenicol resistance.

This analysis revealed that tetracycline resistance was present in all Inaba serotype strains which contained SXT ICE and which were isolated during the period 2015 to 2017. This observation led us to examine whether antibiotic selection pressure played any role in serotype switching. Therefore, we gleaned trends from the results of antibiotic sensitivity tests for tetracycline as shown in Fig. S3 for the period 2005 to 2018. It was observed that, beginning from 2012, tetracycline resistance was observed in more than 90% of Ogawa serotype isolates tested during the period 2012 to 2015. The same was observed in Inaba serotype cases from the 2 years that followed (2016 and 2017). These results corroborated the antibiotic gene repertoire of SXT ICE determined in Ogawa and Inaba serotype strains of clade B isolated from 2015 to 2017. Remarkably, all Ogawa serotype cases in 2018 were susceptible to tetracycline and this was also in agreement with our observations that clade A Ogawa serotype isolates contained different SXT ICEs and did not harbor a *tet*(59) gene, except for strain NALMLE05, which was susceptible to tetracycline but carried this gene in its genome and belonged to clade B.

10.1128/mBio.03339-19.3 FIG S3 Temporal distribution of tetracycline resistance in Vibrio cholerae serotypes. The distribution of tetracycline resistance among Ogawa and Inaba serotypes of V. cholerae for a 14-year period (2005 to 2018) as tested at icddr,b, Dhaka, Bangladesh. The Ogawa and Inaba serotypes of V. cholerae are indicated in blue and red, respectively. Download FIG S3, TIF file, 0.8 MB. Copyright © 2020 Baddam et al. 2020 Baddam et al. This content is distributed under the terms of the Creative Commons Attribution 4.0 International license.

## DISCUSSION

Beginning from the year 2016, as per our data, there appears to have been a sudden surge in the number of Inaba cases as reported during seasonal outbreaks and this continued until the end of 2017. To examine this scenario in the backdrop of serotype distributions of previous years, we first analyzed the trends of metadata of a 14-year period, from 2005 to 2018. This led to identification of differing levels of predominance of serotypes at different intervals as shown in Fig. 1, although the Ogawa serotype was identified as the dominant cause of cholera for most of the period. The predominance of Inaba in 2016 to 2017 was observed after nearly a decade where increase in the rate of Inaba cases first appeared in 2006. A similar trend was observed in India (during 1989 to 1999) according to a previous report (17). Further, an increase in Inaba cases in 2006 was also identified in a zone of cholera endemicity (Kolkata, India) in a previous study which analyzed serotype distribution from 2003 to 2011 (22).

Here, we present whole-genome sequencing data of 34 multidrug-resistant V. cholerae O1 strains belonging to both serotypes isolated during 2015 to 2018. Genomic characterization of these isolates in great detail revealed distinct differences in the composition of mobile genetic elements. Furthermore, a whole-genome-based phylogenetic tree was built to determine the genetic relatedness of the Inaba serotype strains which emerged in 2016 to 2017 in relation to prevailing Ogawa serotype strains in both the preceding and following years. As shown in Fig. 2, the tree revealed high genetic similarity among 34 sequenced isolates in the present study and that they differed from the reference genome of strain N16961 by only less than 160 SNPs. This observation appeared to be in agreement with a previous genomic report that identified a low mutation rate for isolates of V. cholerae of the seventh pandemic (5).

Although it appears that the clonal expansion of Inaba isolates in clade B is responsible for the peaks of infection during 2016 to 2017, their progenitor strains could have been the Ogawa serotype strains in 2015, which coclustered in the same clade and shared similar mobilome. This supposition was further backed by findings indicating disruptions of the *wbeT* gene at identical positions in all Inaba serotype strains isolated during 2016 to 2018. There is a lesser probability, however, for the other scenario, although only a single Inaba serotype strain (NALMLE45) was sequenced from 2015 which had a disruption at a different location in the *wbeT* gene. Altogether, it appears that the switching to Inaba serotype in 2016 to 2017 was beneficial to V. cholerae for evading the immune defenses and enabling easy persistence during this period and that this was perhaps achieved by disruption of the *wbeT* gene through a transposable element similar to the one identified in a previous study on serotype switching of this organism (22). However, in contrast to that previous report, we did not find any single-base substitutions in the *wbeT* gene that led to inactivation of this gene. Also, no mutations responsible for the reversal from the Inaba serotype to the Ogawa serotype were identified.

A distinct clade (clade A) of Ogawa isolates that started predominating from 2018 also contained different composition of mobilome and *ctxB* genotype when compared to those of other clade B isolates as shown in Fig. 2. The genetic changes in the *ctxB* gene encoding the B subunit of CT have played an important role in the evolution of V. cholerae. The classical genotype of *ctxB* (*ctxB1*) and the El Tor allele of *rstR* (repressor gene of CTX phage)—genetic traits specific to early wave 3 isolates of the seventh pandemic—were mainly observed in isolates of clade B. The clade A Ogawa serotype isolates of 2018 mainly contained the *ctxB7* allele (His20 replaced by Asn) along with *rstR*^El Tor^, which is unique to the current wave 3 isolates of the seventh pandemic (28). Taking the results together, the Inaba serotype isolates contained mainly classical *ctxB* alleles but the *ctxB7* allele was more prevalent in Ogawa serotype isolates. Such a biased presence of the *ctxB* allele among Ogawa and Inaba serotypes was reported earlier (29). Furthermore, it was reported previously that the null mutation in the *rtxA* gene may form the genetic background for subsequent emergence of *ctxB7* allele (25, 30) as it is observed in the case of clade A Ogawa isolates of this study. The persistence of clade B Inaba serotype isolates was also noted in the year 2018, and majority of them lacked the SXT ICE. This kind of coexistence of strains belonging to both serotypes from closely related clades—clade A and clade B—during a particular outbreak is possible in a setting of cholera endemicity such as Dhaka, Bangladesh. However, variations at the level of penetrance/contribution to the outbreak might exist among clades as shown in Fig. 1 for the year 2018.

Remarkably, distinct variations between the two clades were observed with respect to mobile elements such as VSP-II, SXT ICE, and PLE1, all of which in general contribute to the pathogenicity and adaptation of V. cholerae (11, 27, 31). The genetic rearrangements in the VSP-II island and in types VSP-IIB and VSP-IIC identified mainly in Ogawa isolates of this study have been previously reported (32). The VSP-IIA type, whose occurrence was mainly observed in Inaba isolates, was not reported earlier, to our knowledge. The differences in SXT ICE elements of this isolate collection also can clearly differentiate the two clades identified in Fig. 2. Furthermore, notable differences in antibiotic resistance may point towards a role of antibiotic selection pressure in serotype switching.

Previous reports have shown that phages also play a role in seasonality of cholera outbreaks, and the selection pressure imposed by them may favor phage-resistant strains compared to the vulnerable ones (33). The PLE mobile element contributes to phage resistance by influencing phage replication and bacterial cell lysis upon infection in order to prevent further propagation of phages (34). However, phages encoding a CRISPR-Cas system with diverse spacers that can inactivate PLEs by targeting different regions of this element have been identified in the previous studies (35). The variable presence of the PLE1 element in isolates of clade B and its complete absence in clade A may represent the dynamism in the coevolution of V. cholerae and its phages, and a role of this element in serotype switching cannot be ruled out. Although serotype distribution was not reported, the presence of PLE1 in V. cholerae strains isolated during different cholera epidemics in Bangladesh from 2011 to 2015 was noted in a previous study (35).

In conclusion, the results of this study suggest that various selective forces such as antibiotic resistance and phage resistance and consequent clonal expansion might contribute to the predominance of a particular V. cholerae serotype during a particular time period. Also, immune pressure might play a role in switching of these serotypes since host immunity induced by a particular serotype would be present if the serotype had been in long term circulation in the population. Furthermore, the impact of environmental factors on the viability of these two serotypes is also not known. The results of the current study highlight the importance of characterizing the mobilomes of V. cholerae genomes in greater detail in order to understand the selective forces behind serotype switching. Although the resolution provided by applying WGS has provided some clues about the dynamics of V. cholerae serotype predominance, the study was limited by the number of representative clinical strains isolated during the transition period and did not include any strains from environment.

## MATERIALS AND METHODS

### Bacterial isolates.

A total of 34 Vibrio cholerae isolates cultured during the years 2015 to 2018 by the Clinical Microbiology and Immunology Laboratory of the icddr,b were selected for whole-genome sequencing. The associated metadata are listed in Table 1. The study did not involve any work on human subjects as such or did not use any identifiable human subject information except the use of alphanumeric codes linked to laboratory isolates. Hence, it was not considered to require a dedicated form of ethics approval. Further, the study benefited from ongoing surveillance protocols/activities (hospital/laboratory/genomics) entailing bacterial agents or their traits such as antimicrobial resistance. All international biosafety practices at biosafety level 2 were observed as applicable and under the advice of the icddr,b Biosafety Office. Before performing whole-genome sequencing, we reconfirmed all 34 isolates following standard microbiological methods according to Clinical & Laboratory Standards Institute (CLSI) guidelines (36). Slide agglutination tests performed with polyvalent and specific antisera (Denka Seiken, Japan) were carried out to differentiate the Ogawa and Inaba serotypes. Agglutination of chicken erythrocytes and polymyxin B susceptibility tests were carried out to confirm biotypes.

### Antibiotic sensitivity testing.

The Kirby-Bauer disk diffusion technique was applied to perform the antibiotic susceptibility testing according to the CLSI guidelines (36). The following 14 commercial antimicrobial agents (Oxoid, USA) were tested against all 34 V. cholerae isolates: ampicillin, cefotaxime, streptomycin, chloramphenicol, erythromycin, azithromycin, co-trimoxazole, tetracycline, nalidixic acid, norfloxacin, ciprofloxacin, nitrofurantoin, polymyxin B, and furazolidone. Escherichia coli ATCC 25922 was used for quality control for the Kirby-Bauer disk diffusion tests. Isolates which exhibited resistance to at least three or more antibiotic classes were considered multidrug-resistant (MDR) isolates. Further, isolates with an intermediate zone of inhibition were also considered resistant.

### Genomic DNA extraction and whole-genome sequencing.

Total bacterial genomic DNA was isolated from a pure broth culture of V. cholerae strains by the use of a Maxwell RSC culture cell DNA extraction kit (Promega, USA) in accordance with the manufacturer’s instructions. DNA samples were quantified using a Quantus Fluorometer (Qubit) in order to check for the quality requirements of samples for sequencing. The whole-genome sequencing was carried out at the Genomics Centre of the International Centre for Diarrheal Disease Research, Bangladesh (icddr,b), using Illumina MiSeq. The sequencing libraries were prepared from 1 ng of genomic DNA using a Nextera XT DNA library preparation kit (Illumina, USA) according to the manufacturer’s instructions. Paired-end sequencing reads (300 bp) were generated using Illumina MiSeq (V3 chemistry kit).

### Genome sequence analysis.

The paired-end read data obtained after whole-genome sequencing were filtered for high-quality reads using Trimmomatic v0.36 (37) and were later *de novo* assembled using SPAdes Assembler v3.10.1 (38). Prokka v1.12 (39) was used for the annotation of resulting assemblies with the ‘–usegenus’ argument, and a customized *Vibrio* genus database was generated for this purpose using genomes downloaded from NCBI. The genome statistics were derived using Artemis (40). The sequence types (ST) were gleaned using MLST tool v2.0.1 (available at https://cge.cbs.dtu.dk/services/MLST).

### Variant calling and pseudogenome generation.

The paired-end sequence reads of each sample were aligned to the reference genome of V. cholerae O1 El Tor strain N16961 (GenBank accession numbers LT907989 and LT907990) using SMALT v0.7.4.4 (ftp://ftp.sanger.ac.uk/pub/resources/software/smalt/). The variant calling was done using samtools mpileup v1.3.1 (41) and bcftools v1.3.1 (http://samtools.github.io/bcftools/) to produce a VCF (variant call format) file of all variants. The single nucleotide polymorphisms (SNPs) identified in the VCF file were filtered for the following metrics—quality (QUAL > 50), map quality (MQ > 30), and mapping depth (DP ≥ 4). Further, for each SNP to be considered of high quality, the number of reads supporting alternative alleles had to be greater than or equal to 75% of the total number of reads aligned at that position; also, of the minimum total of 4 reads at each site, at least 2 had to map to each strand.

A pseudogenome was built for each sample by replacement with the alternative allele at variant sites in the reference genome. Further, those sites with variants which did not pass filtering performed with the metrics described above were replaced by “N” and indels (insertions and deletions) identified with respect to the reference genome were ignored. Thus, the pseudogenome generated for each sample had the same length as that of reference genome; this way, we generated a multi-FASTA whole-genome alignment of 34 in-house-sequenced samples. The M66 prepandemic strain was included in the alignment using Snippy v3.2 (https://github.com/tseemann/snippy) with a ‘–ctgs’ flag as the genome sequence was available as a contig file. The alignment was supplied as an input to Gubbins v2.3.4 (42) in order to identify putative recombination regions, and the filtered polymorphic sites thus obtained outside recombination were thereafter used for phylogeny construction. The pairwise SNP distances for 34 in-house-sequenced isolates with respect to the reference genome of strain N16961 were obtained from the alignment using snp-dists v0.6.3 (https://github.com/tseemann/snp-dists).

Roary (43) was used with prokka annotated assemblies to determine the pan-genome of in-house-sequenced Vibrio cholerae strains. The core genes identified without paralogs were aligned using PRANK (44), and gaps were removed from the alignment using trimAI (45). In order to determine the phylogenetic relatedness of these strains, SNPs from the alignment were gleaned using snp-sites (46).

### Phylogeny.

RaxML (47) was used to construct phylogenetic trees by utilizing multi-FASTA nucleotide alignments (whole genome and core genome) generated as described above. The GTR nucleotide substitution model with gamma correction was applied using 500 bootstrap replicates. The trees thus built were visualized and annotated with various information using iTOL (48).

### Genetic analysis.

The status of the wbeO1 encoding the O1 antigen (49) (GenBank accession: KC152957.1) and that of the *wbeT* gene (14) (GenBank accession no JF284685) responsible for determination of the serotype were analyzed using BLASTn (50). Further, in the case of Inaba serotype isolates, although only fragments of a transposable element similar to a SXT IS4 family transposase gene (gb|KC709654.1:577-1965) were identified using BLASTn in the genome assemblies of isolates (usually mapped to the end region of contigs harboring a disrupted *wbeT* gene), further confirmation of this gene disruption was obtained by applying the ISmapper v0.1.5.2 (51) approach with paired-end read data. SXT elements in the genomes were identified using an approach similar to that described previously (52). Briefly, first, the contigs harboring the truncated *prfC* gene due to insertion at the 5′ end were identified as part of the SXT element. Second, as the downstream integrase gene showed high similarity to the genome of V. cholerae strain E4, all contigs of each genome were rearranged according to it using Contig Layout Authenticator (53) in order to identify the neighboring SXT-like contigs. Thus, collected SXT-like sequences were joined to identify the closest match available in the public domain using BLASTN. The antibiotic resistance genes were identified using the CARD resistance database (54). The variations in the VSP-II island were identified using BLASTn, considering the sequence of the 26.9-kbp genomic island marked in the genomic sequence of V. cholerae strain N16961. The GView Web server (https://server.gview.ca/) was used to generate comparisons of VSP-II island and SXT ICE elements. All the genomes were screened for PLE1 and PLE2 sequences using BLASTn. Also, the genetic variations in important virulence genes—*ctxB*, *tcpA*, and *rtxA*—were identified using BLASTn.

### Data availability.

The genome sequence data generated as part of this study have been deposited in NCBI under BioProject identifier (ID) PRJNA523111, and accession numbers of individual genomes are listed in Table 1.
