# Supplementary figures and images for: Genome Dynamics of Vibrio cholerae Isolates Linked to Seasonal Outbreaks of Cholera in Dhaka, Bangladesh
Source: mBio. 2020 Feb 11;11(1):e03339-19. doi: 10.1128/mBio.03339-19 (PMC7018647; doi:10.1128/mBio.03339-19)

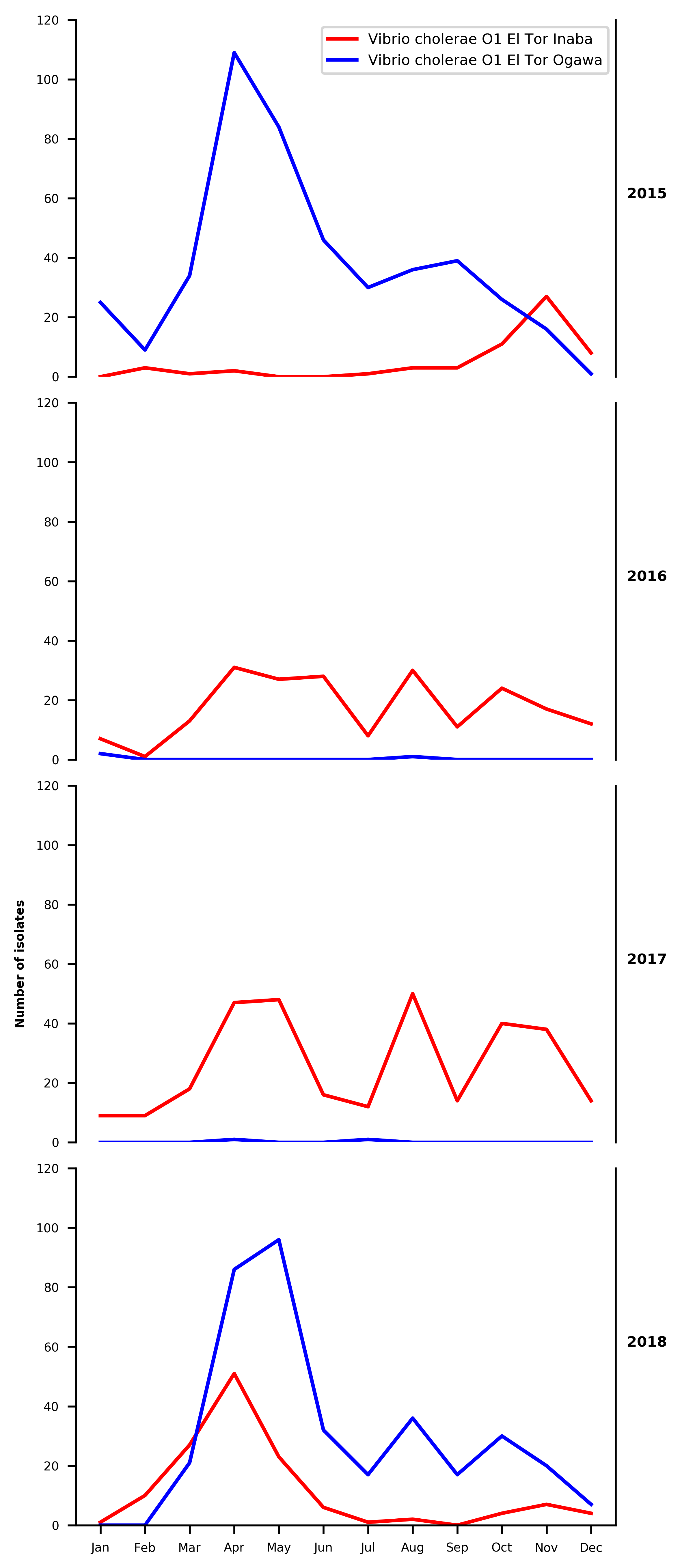

Supplement: FIG S1 [file mBio.03339-19-sf001.tif]

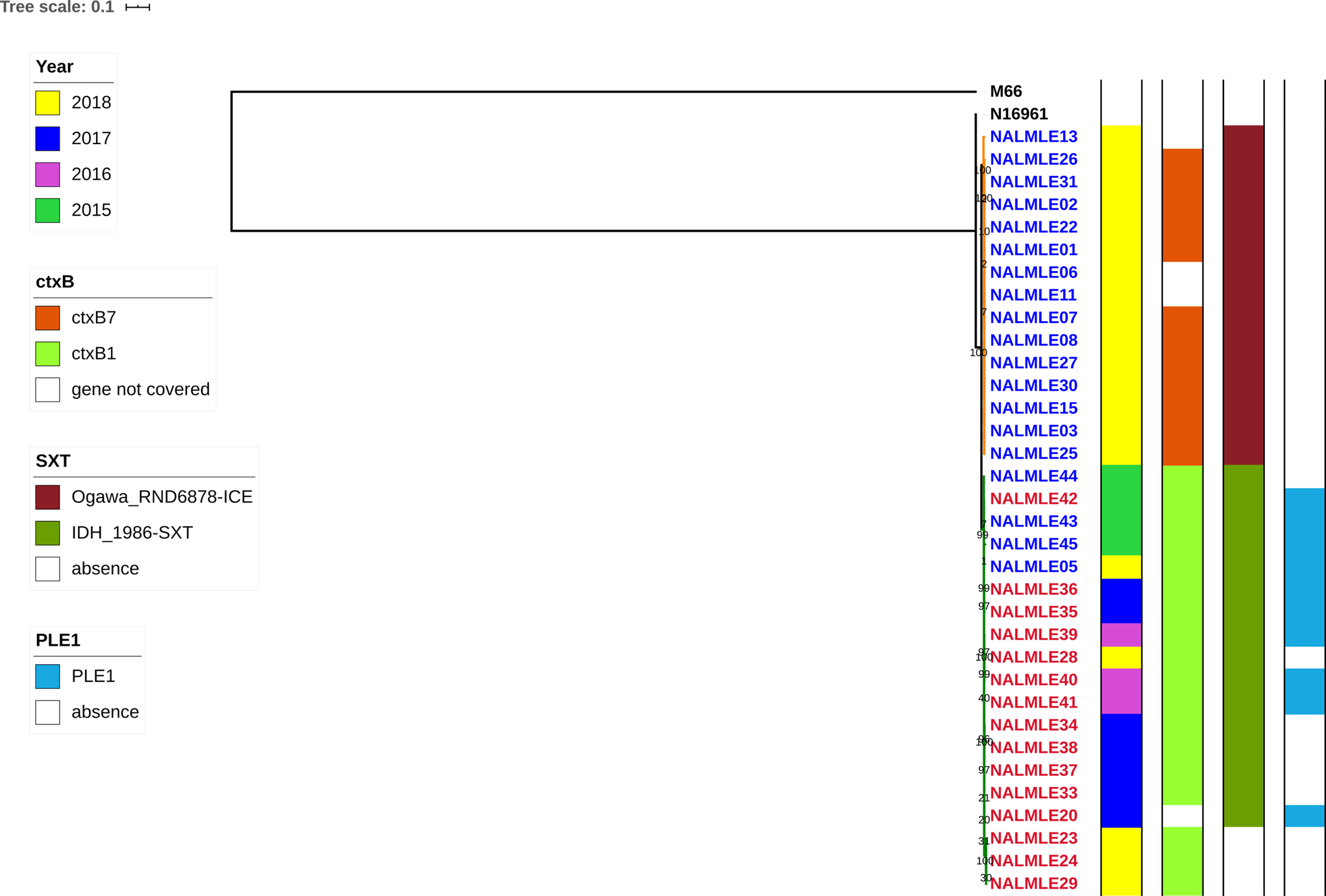

Supplement: FIG S2 [file mBio.03339-19-sf002.tif]

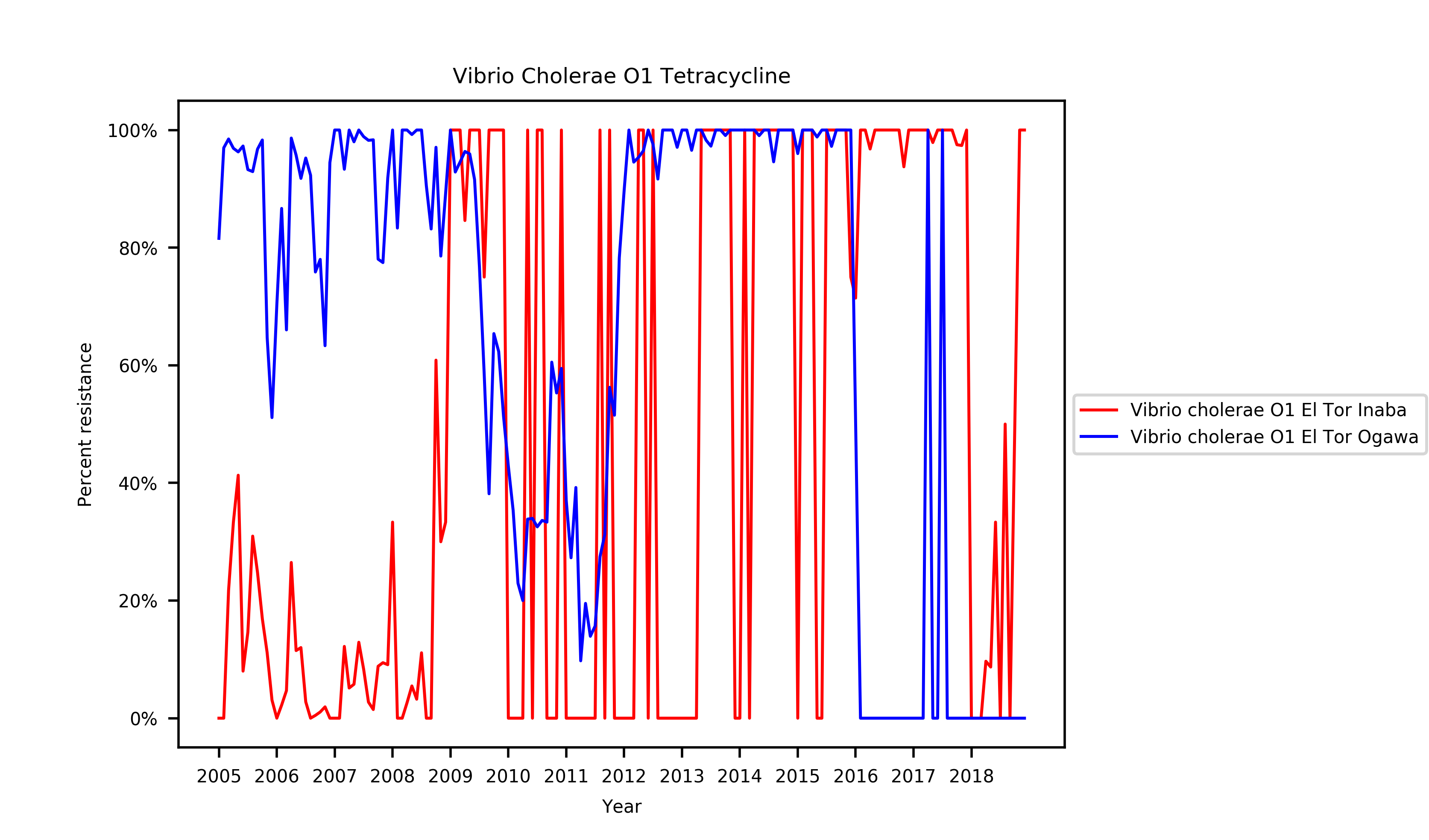

Supplement: FIG S3 [file mBio.03339-19-sf003.tif]
